# Distribution of Hydrogenases in Cyanobacteria: A Phylum-Wide Genomic Survey

**DOI:** 10.3389/fgene.2016.00223

**Published:** 2016-12-27

**Authors:** Vincenzo Puggioni, Sébastien Tempel, Amel Latifi

**Affiliations:** Laboratoire de Chimie Bactérienne UMR 7283, Centre National de la Recherche Scientifique (CNRS), Aix-Marseille UniversityMarseille, France

**Keywords:** cyanobacteria, genomes, hydrogenase, oxygen tolerance

## Abstract

Microbial Molecular hydrogen (H_2_) cycling plays an important role in several ecological niches. Hydrogenases (H_2_ases), enzymes involved in H_2_ metabolism, are of great interest for investigating microbial communities, and producing BioH_2_. To obtain an overall picture of the genetic ability of Cyanobacteria to produce H_2_ases, we conducted a phylum wide analysis of the distribution of the genes encoding these enzymes in 130 cyanobacterial genomes. The concomitant presence of the H_2_ase and genes involved in the maturation process, and that of well-conserved catalytic sites in the enzymes were the three minimal criteria used to classify a strain as being able to produce a functional H_2_ase. The [NiFe] H_2_ases were found to be the only enzymes present in this phylum. Fifty-five strains were found to be potentially able produce the bidirectional Hox enzyme and 33 to produce the uptake (Hup) enzyme. H_2_ metabolism in Cyanobacteria has a broad ecological distribution, since only the genomes of strains collected from the open ocean do not possess *hox* genes. In addition, the presence of H_2_ase was found to increase in the late branching clades of the phylogenetic tree of the species. Surprisingly, five cyanobacterial genomes were found to possess homologs of oxygen tolerant H_2_ases belonging to groups 1, 3b, and 3d. Overall, these data show that H_2_ases are widely distributed, and are therefore probably of great functional importance in Cyanobacteria. The present finding that homologs to oxygen-tolerant H_2_ases are present in this phylum opens new perspectives for applying the process of photosynthesis in the field of H_2_ production.

## Introduction

Microbial hydrogen (H_2_) metabolism is a process that occurs in many different environments. In addition to being a key metabolic factor in several biological communities, H_2_ has attracted considerable interest as a candidate environmentally friendly energy carrier. The use of photosynthetic organisms such as microalgae and cyanobacteria has been tested worldwide for this purpose. In cyanobacteria, the main enzymes involved in H_2_ metabolism are nitrogenases and hydrogenases (H_2_ases) (Reviewed in Bothe et al., 2010). Nitrogenases fix molecular nitrogen (N_2_) and produce H_2_ as a byproduct (D'Eustachio and Hardy, 1964). H_2_ases are metalloprotein enzymes which catalyze in several microorganisms the reversible reaction:

$$\begin{array}{l} 2{\text{H}}^{+}+2{\text{e}}^{-}↔{\text{H}}_{2} \end{array}$$

(for a recent Review see Peters et al., 2015).

They are usually classified into three phylogenetically independent classes: [Fe] H_2_ases, [FeFe] H_2_ases, and [NiFe] H_2_ases (Vignais and Billoud, 2007). Since [Fe] H_2_ases are light-sensitive enzymes (Chen et al., 2002), they can be considered as for limited interest in the context of H_2_ photoproduction. The [FeFe] H_2_ases present in anaerobic bacteria and some phototrophic eukaryotes preferentially catalyze the evolution of H_2_ at high frequencies; these enzymes are also characterized by their high sensitivity to oxygen (O_2_) (Melis et al., 2000; Florin et al., 2001; Winkler et al., 2002; Peters et al., 2015). The [NiFe] H_2_ases, which have been found to exist in Archaea and in several aerobic and anaerobic bacterial phyla, are mainly involved in H_2_ oxidation but can also catalyze the reduction of protons to H_2_ (Vignais and Billoud, 2007). They consist of a large subunit containing the bimetallic center [NiFe] and a small subunit containing [FeS] clusters (Volbeda et al., 1995, 1996; Peters et al., 2015). Based on a phylogenetic analysis of the large subunit, and more specifically, on two highly conserved regions located in this subunit near the [NiFe] center (the L1 and L2 regions), the [NiFe] H_2_ases have been classified into the eight different groups presented in Table 1 (Vignais et al., 2001; Vignais and Billoud, 2007). The maturation of [NiFe] H_2_ases involves six proteins (HypFCDEAB), which synthesize the non-protein ligands (CO and CN) and assemble the active site (Dernedde et al., 1996; Hansel et al., 2001; Hoffmann et al., 2006). In the last step in the process of biosynthesis, the C terminal part of the large subunit is cleaved by a specific peptidase (Thiemermann et al., 1996; Devine et al., 2009).

**Table 1:** **Overview of the main features of [NiFe] H_2_ases**.

**Group**	**Name**	**Function**	**H_2_**	**O_2_sensitive/resistant**	**References**
1	Membrane bound H_2_ uptake H_2_ases	H_2_ uptake under aerobic and/or anaerobic conditions.	Oxidation	Sensitive and Resistant	Higuchi et al., 1999; Marques et al., 2010; Dementin et al., 2011
2a	Cyanobacterial uptake H_2_ases	Uptake of H_2_ produced by nitrogenase.	Oxidation	Sensitive	Oxelfelt et al., 1998; Tamagnini et al., 2007; Zhang et al., 2014
2b	H_2_-signaling H_2_ases	H_2_ perception and signaling.	Oxidation	Resistant	Buhrke et al., 2004, 2005; Roncaroli et al., 2015
3a	F_420_-reducing H_2_ases	H_2_ utilization during methagenosis.	Oxidation evolution	Sensitive	Hendrickson and Leigh, 2008; Vitt et al., 2014
3b	Tetrameric bifunctional H_2_ases	Regulation and redox balance.	Oxidation evolution	Sensitive and resistant	Bryant and Adams, 1989; Jenney and Adams, 2008; Berney et al., 2014
3c	Methyl-viologen-reducing H_2_ases	H_2_ uptake during methagenosis.	Oxidation	Sensitive	Kaster et al., 2011
3d	Soluble bidirectional H_2_ases	Regulation and redox balance.	Oxidation evolution	Sensitive and resistant	McIntosh et al., 2011; Lauterbach and Lenz, 2013
4	H_2_-evolving, energy-conserving, membrane-associated H_2_ases	Coupling of formate or carbon monoxide to H_2_ evolution.	Evolution	Sensitive	Bagramyan et al., 2002; McDowall et al., 2014
5	Actinobacteria [NiFe]-H_2_ases	H_2_ uptake during starvation.	Oxidation	Resistant	Schäfer et al., 2013

Although the activity of most of the [NiFe] H_2_ases tends to be inhibited by O_2_, some members of this class remain active in the presence of O_2_ and have therefore been called O_2_-tolerant. The O_2_-tolerant H_2_ases described for the first time in the anoxigenic bacterium *Rubrivivax gelatinosus* (Maness et al., 2002) occur in the Group 1 membrane-bound H_2_ases (MBH), the H_2_-signaling group (RH, Group 2b) (Buhrke et al., 2005; Duché et al., 2005), the tetrameric bifunctional H_2_ases (group 3b) (Jenney and Adams, 2008; Kwan et al., 2015), the bidirectional H_2_ases (group 3d) (Horch et al., 2013; Karstens et al., 2015) and the recently identified Group 5 Actinobacterial-H_2_ases (Table 2) (Constant et al., 2010; Lubitz et al., 2014). In the case of the MBH enzymes, the main difference between the standard and tolerant members focuses on the [FeS] cluster located near the [NiFe] site. Instead of the canonical [4Fe4S] present in the standard enzymes, a [4Fe3S] cluster coordinated by six cysteine residues occurs in the tolerant enzymes (Pandelia et al., 2011; Shomura et al., 2011). This proximal [4Fe3S] is the most striking feature thought to be linked to O_2_-tolerance (Goris et al., 2011; Lukey et al., 2011). The O_2_-insensitivity of the RH-H_2_ases of *Ralstonia eutropha* H16 depends on the size and shape of the intramolecular hydrophobic cavity giving access to the active [NiFe] site (Buhrke et al., 2005). The molecular mechanism underlying the O_2_-tolerance of the Group 3 SH enzymes and that of the actinobacterial H_2_ases still remains to be elucidated.

**Table 2:** **Overview of the main features of O_2_-tolerant H_2_ases in several organisms**.

**Group**	**Name**	**Cluster Fe-S small subunit**	**Structural basis of O_2_-tolerance**	**Example**	**References**	**Homolog in cyanobacteria**
1	Membrane bound H_2_ uptake H_2_ases (MBH)	p [4Fe3S] m [3Fe4S] d[4Fe4S]	Transfer electron from the proximal cluster to active site to reduce O_2_ to water.	*Rubrivivax gelatinosus*, Hyd-1 *Escherichia coli*	Maness et al., 2002; Evans et al., 2013	*Lyngbya confervoides* BDU141951
2b	H_2_-signaling H_2_ases (RH)	p [4Fe4S] m [4Fe4S] d[4Fe4S]	The gas channel is narrower than standard H_2_ases and the O_2_ cannot interact with the active site.	*Rhodobacter capsulatus, Ralstonia eutropha*	Buhrke et al., 2005; Duché et al., 2005	None
3b	Tetrameric bifunctional H_2_ases (PfSHI)	p [4Fe4S] m [2Fe2S] d[4Fe4S]	No formation of the slowly reactivating state Ni-A	*Pyrococcus furiosus*	Jenney and Adams, 2008; Kwan et al., 2015	*Cyanothece* sp. PCC 7425, *Leptolyngbya boryana* PCC 6306, *Mastigocoleus testarum* BC008
3d	Soluble bidirectional H_2_ases (ReSH)	[4Fe4S]	Reduction of O_2_ in water. Cys39 and Trp42 are demonstrated important for O_2_ tolerance	*Ralstonia eutropha*	Horch et al., 2013; Karstens et al., 2015	*Aphanocapsa montana* BDHKU210001
5	Actinobacteria [NiFe]-H_2_ases (AH)	p [4Fe4S] m [4Fe4S] d[4Fe4S]	Unknown	*Streptomyces avermitilis, Ralstonia eutropha*	Constant et al., 2010; Lubitz et al., 2014	None

Cyanobacteria, the only prokaryotes capable of oxygenic photosynthesis, form a large and morphologically diverse bacterial group consisting of five morphological subsections. The unicellular organisms that undergo binary fission belong to subsection I (*Chroococcales*). The unicellular strains that divide through multiple fission processes form subsection II (*Pleurocapsales*), and subsection III consists of filamentous strains which are unable to perform cell differentiation (*Oscillatoriales*). The strains in subsections IV and V are filamentous and able to differentiate specific cells called heterocysts, which are dedicated to N_2_ fixation (Rippka et al., 1979). Cyanobacteria are widely distributed in various environments (from oceans to desert crusts), where they contribute importantly to primary production and N_2_ fixation processes (Garcia-Pichel et al., 2003). N_2_-fixation in these organisms is mainly achieved by a molybdenum-iron ([MoFe]) nitrogenase which consists of two subunits, a Fe-protein encoded by *nifH*, and a Mo-Fe protein encoded by *nifDK* genes (Smith and Eady, 1992). The maturation process requires three essential (*nifBEN*) and three no essential genes (*nifUSV*) (Reviewed in: Rubio and Ludden, 2008). The reduction of N_2_ is accompanied by the formation of H_2_ (Berman-Frank et al., 2003). Cyanobacteria contain two different [NiFe] H_2_ases: the bidirectional [NiFe] H_2_ase (Hox, Group 3d) and the uptake H_2_ase (Hup, Group 2a) (Tamagnini et al., 2007). The Hup H_2_ase is a heterodimeric enzyme encoded by the *hupSL* genes, which consumes the H_2_ produced by the nitrogenase (Houchins and Burris, 1981; Lindblad and Sellstedt, 1990). The bidirectional Hox H_2_ase, which can oxidize H_2_ and reduce H^+^, can exist in both diazotrophic and non-diazotrophic strains, and is thought to be a heteropentameric enzyme encoded by *hoxEFUYH* genes (Schmitz et al., 1995). In the unicellular cyanobacterium *Synechocystis* PCC 6803, the bidirectional H_2_ase has been shown to be essential under mixotrophic and nitrate limiting conditions, which suggests that this enzyme functions as electron sink for reduced flavodoxin/ferredoxin (Gutekunst et al., 2014). The ability of the Hox enzymes to be quickly reactivated after being inhibited by O_2_ has made them the most frequently used H_2_ase in studies on H_2_ production in cyanobacteria (Serebryakova et al., 1996; Germer et al., 2009; McIntosh et al., 2011). The main limitations of using the cyanobacterial Hox enzymes in large scale H_2_ production processes are the low levels of H_2_ produced and the fast reversal of the enzymatic reaction into oxidation (Tamagnini et al., 2007; Rögner, 2013). During the last decade, genetic engineering approaches were used in several studies in order to overcome these technological barriers with a relative success (Masukawa et al., 2002; McNeely et al., 2010; Baebprasert et al., 2011; Ortega-Ramos et al., 2014; Nyberg et al., 2015). Cyanobacterial strains and/or genomes have also been widely explored in order to unravel the complex picture of H_2_ases (Ludwig et al., 2006; Barz et al., 2010; Kothari et al., 2012, 2013). These studies have opened new perspectives, since they have shed light on the H_2_ production potential of strains other than those previously used as laboratory models. Since the publication of these studies, larger numbers of cyanobacterial genomes have been sequenced, which has greatly improved the genomic coverage of all the phylum (Shih et al., 2013). In order to investigate cyanobacterial H_2_ metabolism more closely, we performed a large-scale analysis of H_2_ases genes distribution in cyanobacteria, which consisted in searching for the genes encoding H_2_ases and the proteins required for their maturation in 130 cyanobacterial genomes. The distribution of H_2_ases in the cyanobacterial phylum inhabiting various environments is discussed.

## Results

### Distribution of H_2_ase encoding genes and of genes involved in their maturation process

Our genomic search for genes encoding H_2_ase and the proteins involved in their maturation helped to complete the picture of which strains may possibly synthesize functional H_2_ase.

**Figure 1:**
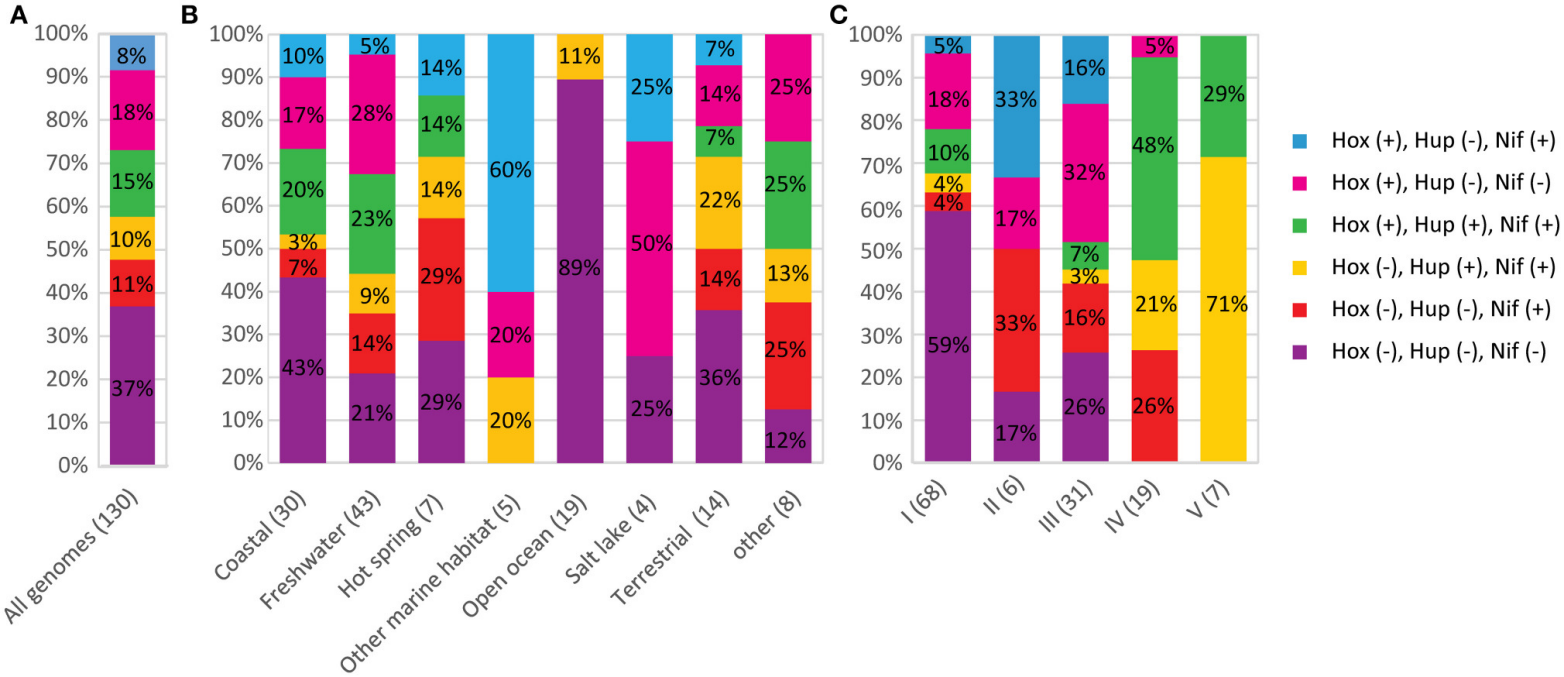
**Co-occurrence of H_2_ases and nitrogenase in cyanobacteria**. The number of genomes analyzed in each graph is shown in parentheses. The various combinations of *hox, hup, nif* genes are presented in different colors. The symbols (+) and (–) indicate that the genes are present or absent respectively. **(A)** Co-occurrence of H_2_ase and nitrogenase encoding genes in the 130 genomes analyzed in this study. **(B)** Distribution of H_2_ase and nitrogenase-encoding genes depending on the habitat of the strains. **(C)** Distribution of H_2_ase and nitrogenase-encoding genes depending on the morphological classification of the strains.

A phylum-wide analysis of the genomic distribution of H_2_ase genes among the cyanobacterial genomes in the CyanoGEBA dataset (Shih et al., 2013) showed that only [NiFe] H_2_ases are present in these organisms. No obvious homologs of [FeFe] or [Fe] H_2_ases were identified. We assumed that only genomes possessing all the *hox* and *hup* genes carry a complete set of H_2_ase-encoding genes. A complete set of H_2_ase-encoding genes was deciphered in 52% of the genomes studied (Figure 1A). Among the 130 genomes analyzed, 49 did not show any H_2_ase-encoding genes (Figure 1A), and 13 genomes did not present the complete set of genes required to encode a functional H_2_ase (Supplementary Table 1). The lack of H_2_ase genes may be attributable to the bacterial habitat, since the proportions of H_2_ases-free genomes differ from one ecological niche to another: the highest proportion of H_2_ase-free strains was detected in the open ocean (89%), and the remaining 11% carried only *hup* genes, which suggests that the cyanobacterial contribution to H_2_ production in the open ocean is negligible (Figure 1B). The distribution of H_2_ase genes and of genes required for their maturation was found to vary in the cyanobacterial phylum, but all the organisms belonging to subsections IV and V have a complete set of genes encoding H_2_ases. H_2_ oxidation and H^+^ reduction activities seem to be generally conserved in these species (Figure 1C). Since the uptake H_2_ase is involved in functional nitrogenase processes, the co-occurrence of H_2_ases, and nitrogenase in various environments was investigated by studying the distribution of [FeMo] nitrogenase structural genes (*nifH and nifDK*), the *nifBEN*, and the *nifUSV* genes involved in the synthesis of the [FeMo]-cofactor synthesis. The *nifH, nifDK*, and *nifBEN* genes were found in all the cyanobacteria genomes (Supplementary Table 2). The *nifBEN* genes were found in co-occurrence with *nifUSV* genes except is six genomes (Supplementary Table 2). Since the *nifSU* genes have been reported to be dispensable in *Anabaena variabilis* (Lyons and Thiel, 1995), one might conclude that their absence does not necessarily mean that the strain is not able to fix nitrogen. It is therefore concluded that all the strains listed in Supplementary Table 2, and whose genomes contain *nifH, nifDK*, and *nifBEN* genes are potentially nitrogen-fixing.

The data obtained here, indicate that *nif* genes are present in genomes harboring *hup* (10%) or *hox* (8%), or both (15%). Eleven percent of the genomes possess *nifH* and *nifDK* without harboring the *hox* and *hup* genes (Figure 1A). The co-occurrence of *nif* and *hup* genes seems to be significantly more frequent in the genomes of strains belonging to subsection V (Figure 1C).

**Figure 2:**
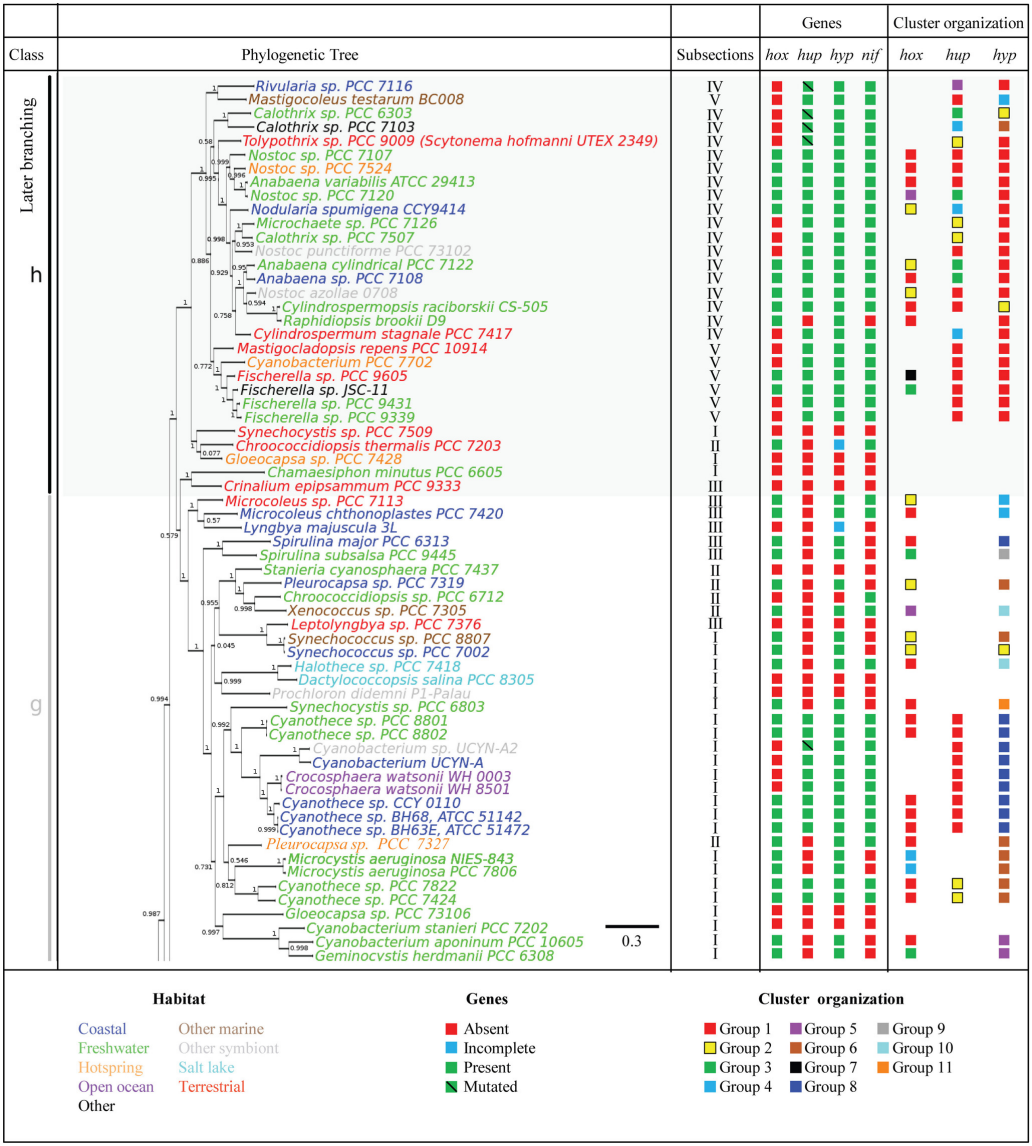
**Phylogenetic distribution of H_2_ases, and nitrogenase in Cyanobacteria**. The species tree used in this study is shown in the left panel. The tree was rooted using the sequences of four outgroup organisms (See Section Methods). The genomes are shown in different colors depending on the habitat of the strains. The presence or absence of selected genes is indicated by green and red squares, respectively. The blue square indicates genomes where the set of *hyp, hup*, or *hox* genes is incomplete (See Supplementary Table 1 for details). The green barred square indicates genetic polymorphism in catalytic residues. The cluster arrangement of *hup, hox*, and *hyp* genes shown in Figure 4 is summarized in the right panel of this picture.

**Figure 3:**
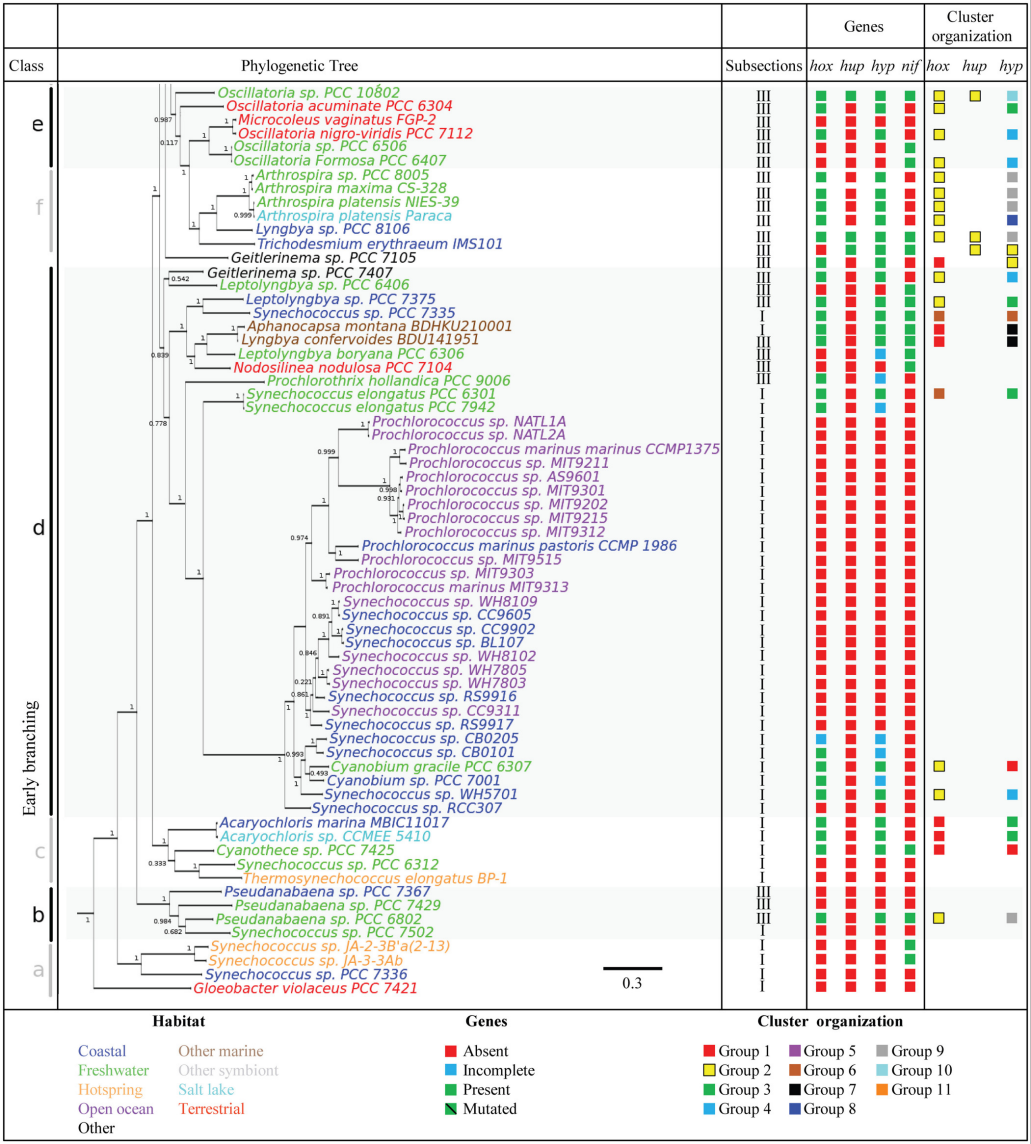
**Phylogenetic distribution of H_2_ases, and nitrogenase in Cyanobacteria**. The species tree used in this study is shown in the left panel. The tree was rooted using the sequences of four outgroup organisms (See Section Methods). The genomes are shown in different colors depending on the habitat of the strains. The presence or absence of selected genes is indicated by green and red squares, respectively. The blue square indicates genomes where the set of *hyp, hup*, or *hox* genes is incomplete (See Supplementary Table 1 for details). The green barred square indicates genetic polymorphism in catalytic residues. The cluster arrangement of *hup, hox*, and *hyp* genes shown in Figure 4 is summarized in the right panel of this picture.

To further assess the distribution of genes encoding H_2_ases among the cyanobacterial phylum, a phylogenetic tree was constructed using 21 concatenated sequences corresponding to the 130 cyanobacterial proteins listed in Supplementary Table 3 (see Methods Section). The *hox* and *hup* genes were found to occur more frequently in the late branches of the tree, although the distribution of *hox* is patchier (Figures 2, 3). The presence of *hyp* genes was always associated with that of at least one of the Hox or Hup sequences, and these genes therefore occur less frequently in the early branching clades, which is clearly illustrated in the case of clade g (Figures 2, 3). The distribution of the nitrogenase-encoding genes (*nifH* and *nifDK*) is in agreement with the phylogenetic tree previuously presented (Bandyopadhyay et al., 2010). These genes are present in four genomes in the early branches of cyanobacterial evolution: clade a [*Synechococcus* sp. JA-2-3B′a(2–13), *Synechococcus* sp. JA-3-3Ab], clade b (*Pseudanabaena* sp. PCC 6802), clade c (*Cyanothece* sp. PCC 7425), and six genomes of clade d. No *hup* genes were ever detected in these early clades, which suggests that the nitrogenase may function naturally in the absence of uptake H_2_ase. This was previously found to occur in *Synechococcus* sp. and *Cyanothece* PCC 7425, which fix N_2_ under anaerobic conditions (Bandyopadhyay et al., 2011). In the genomes of the strains *Nostocales* and *Stignematales* (subsections V and VI), which belong to clade h, the *nif* and *hup* genes were always found to co-occur (Figures 2, 3). It is also worth noting that the co-occurrence of *hox, hup* and *nif* genes was observed only in the late branches of the tree.

### Distribution, conservation, and physical organization of the *hox* genes

The genes encoding the bidirectional H_2_ases (*hox*Y, *hox*H) and the *hox*U, *hox*E, *and hox*F genes encoding the diaphorase part are widely distributed among the cyanobacterial phylum and are particularly abundant in the genomes of organisms belonging to subsections II, III, and IV (Figures 1B,C and Supplementary Table 4). All the *hox* genes listed in Supplementary Table 4 potentially encode soluble H_2_ases belonging to subgroup 3d (Vignais et al., 2001). In the large subunit (HoxH), the sequences of the L1 and L2 motifs typical of each [NiFe] group show a high level of conservation. Only a few amino-acid substitutions were observed in the L1 motif in seven genomes of strains from various habitats (terrestrial, coastal, and freshwater strains) (Supplementary Table 5). The Cysteine residues involved in the coordination of metal ions are strictly conserved in all the HoxH and HoxY sequences. The three subunits in the diaphorase of the bidirectional H_2_ase part (HoxE, HoxF, and HoxU) also contain the conserved cysteine residues potentially required for the coordination of [2Fe2S] and [4Fe4S] clusters. These cysteine residues are largely conserved, since the only few exceptions observed were HoxF and HoxU proteins in *Synechococcus* sp. CB0205, *P. hollandica* PCC 9006, and *Cyanobium* sp. PCC 7001 (Supplementary Table 1). These genomes also lack some of the genes involved in the maturation process (Supplementary Table 1). The bidirectional H_2_ase in these strains may therefore not be active. The last step in the maturation of the bidirectional H_2_ases involves the HoxW endopeptidase. The co-occurrence of the *hoxW* gene and the *hox* structural genes (HYUEF) was observed in all the genomes analyzed (Supplementary Table 4). Based on the difference between the patterns of expression of the structural *hox* genes and *hoxW*, it has been suggested that the endopeptidase HoxW might have multiple functions in cyanobacteria (Wünschiers et al., 2003). The results of the present study confirm this assumption, since *hoxW* homologs were found to exist in four genomes containing no *hox*YHUEF genes (Supplementary Table 4). In addition, the presence of multicopies of the *hoxW* gene observed in three genomes provides a further argument supporting this hypothesis (Supplementary Table 4).

**Figure 4:**
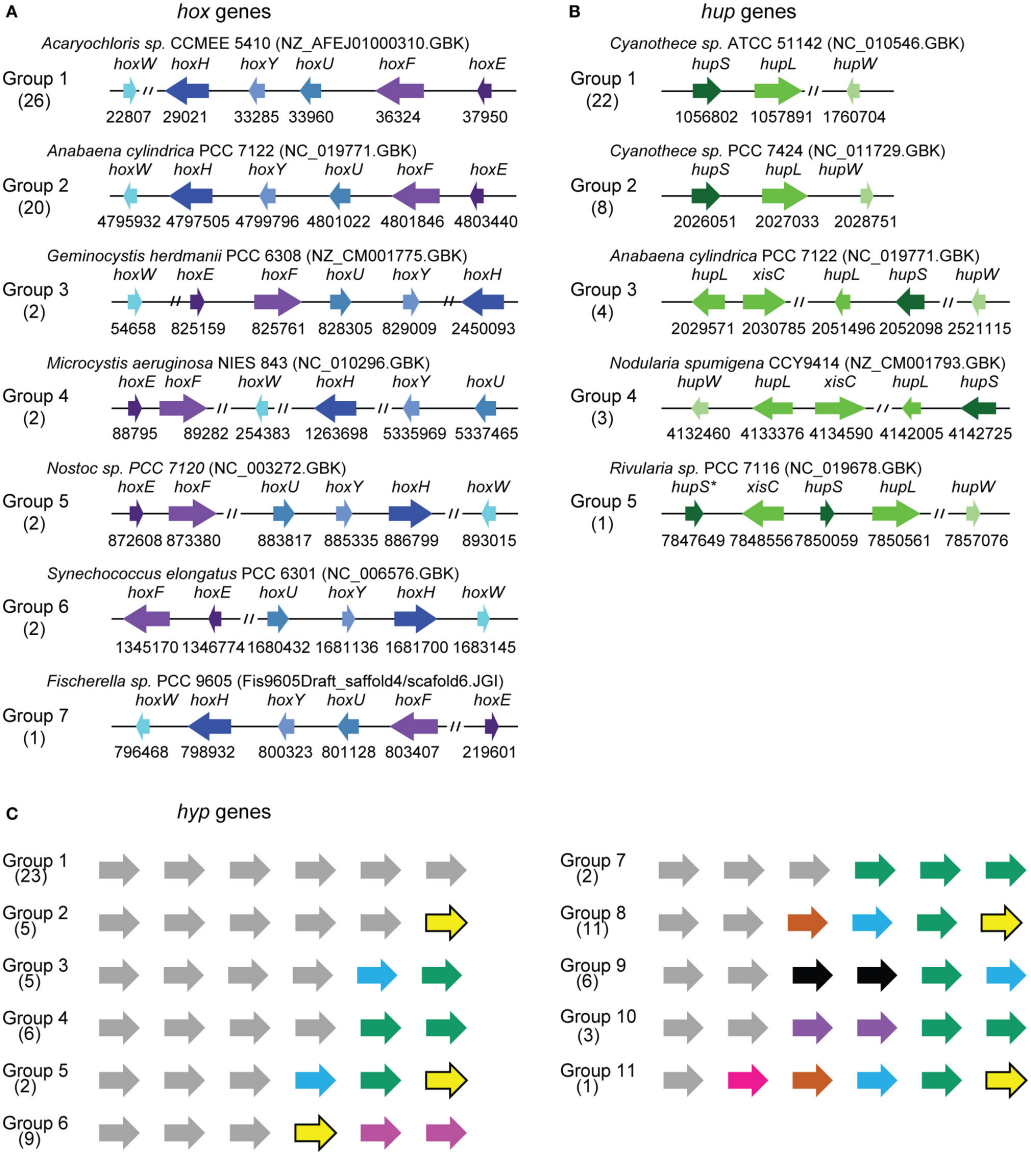
**Representative cluster arrangements of hox, hup and hyp genes in cyanobacterial genomes. (A)** Physical organization of hoxHYDEFWgenes. **(B)** Physical organization of hupSLW genes. **(C)** Physical organization of the hypABCDEF genes. The genes located in the same region are indicated in the same color. The complete physical data on all the genomes studied are presented in Supplementary Figure 1.

Seven different patterns of organization were observed among the structural *hox* genes (Figure 4A, Supplementary Figure 1). In Group 1, the *hoxE, hoxF, hoxU, hoxY*, and *hoxH* genes are clustered together and show the same orientation, whereas the *hoxW* gene occupies another position in the genome. Group 1 includes 26 genomes belonging to all the subsections except subsection V. Group 2 includes 20 genomes belonging to subsections I, II and IV, and all the structural *hox* genes (EFUYHW) are clustered together in the same orientation (Figure 4A). Group 3 contains two genomes belonging to subsections I and V: the *hoxE, hoxF, hoxU, hoxY* are clustered together and in the same orientation, whereas the *hoxW* and *hoxH* are located in another part of the genomes. The *hox* genes are more widely scattered in Groups 4-6: *hoxE* and *hoxF* are clustered together and the other *hox* genes are either clustered or scattered in various combinations. All the hox genes *hoxF, hoxU, hoxY, hoxH*, and *hox W* are clustered together in *Fiscarella* sp. PCC 9605 (Group 7), whereas *hoxE* is located in another part of the genome. The organization of the *hox* genes is generally not conserved throughout the tree of species, where the seven groups are randomly distributed among the eight clades (Figures 2, 3).

### Distribution, conservation, and physical organization of the *hup* genes

HupL and HupS homologs encoding the large and small subunits of the uptake H_2_ase, respectively, were identified only in genomes of diazotrophic strains belonging to subsections I, III, IV, and V (Figure 1C and Supplementary Table 6). The strains carrying uptake H_2_ase genes are widely distributed in various habitats. They are absent only in the genomes of strains collected from salt lakes (Figure 1B). The amino acid sequences of HupS and HupL show a high degree of conservation: the L1 and L2 motifs typical of H_2_ases belonging to group 2a (Vignais and Billoud, 2007) were found to be conserved in all the Hup sequences analyzed. These motifs include the cysteine residues involved in the coordination of the [NiFe] in the case of HupL and [FeS] in that of HupS (Vignais and Billoud, 2007). In the genomes of *Calothrix* sp. PCC 7103, *Tolypothrix* sp. PCC 9009, *Rivularia* sp. PCC 7116, Cyanobacterium sp. UCYN A2, and *Calothrix* sp. PCC 6303, since the HupS sequence shows deletions and substitutions of the residues involved in the binding of [FeS] cluster, these enzymes may be inactive or might have different enzymatic characteristics (Supplementary Figure 2). The specific peptidase HupW was identified in all the genomes carrying *hupSL* genes (Supplementary Table 6). The HupW sequences consistently showed well-conserved residues thought to contribute importantly to the specific interactions between the peptidase and its cognate H_2_ase subunit (Devine et al., 2009).

In all the genomes analyzed, the *hupS* and *hupL* genes form clusters. The organization of the five groups of *hup* genes depends on the location of the *hupW* gene and the disruption (or otherwise) of *hupS* or *hupL* genes by the *xisC* gene (Figure 4B). The distribution of these clustering groups varies in the tree of species (Figures 2, 3). Groups 1 or 2 are mostly present throughout the late branches of the tree (clades f, e, g, and h), whereas groups 3, 4, and 5 occur only in clade h (Figure 4B).

### Distribution, conservation, and physical organization of the *hyp* genes

Almost all the cyanobacterial genomes harboring structural H_2_ase genes (*hox, hup*, or both) also harbor the *hypABCDEF* genes known to encode proteins involved in the maturation of the H_2_ase (Supplementary Table 7), apart from the genomes of *Chroococcidiopsis thermalis* PCC 7203, *Synechococcus elongatus* PCC 7942, *Synechococcus* sp. CB0101, and *Synechococcus* sp. PCC 7336, from which some *hyp* genes are missing. (Supplementary Table 1). Whether the maturation of the H_2_ase in these strains involves different mechanisms, or whether the maturation process is not efficient in these case is still an open question.

Since little is known about the process of H_2_ase maturation in cyanobacteria, we analyzed the amino acid composition of the Hyp proteins in the light of the data available in the literature on other organisms. All the information based on the resolution of the crystal structure of the HypF protein of *Caldanaerobacter subterraneus* (Shomura and Higuchi, 2012), that of the HypECDA of *Thermococcus kodakarensis* (Watanabe et al., 2009, 2012; Tominaga et al., 2013) and that of the HypB of *Archaeoglobus fulgidus, Bradyrhizobium japonicum*, and *Escherichia coli* (Olson and Maier, 2000; Chan et al., 2012; Douglas et al., 2013) are summarized in Supplementary Table 7 and Supplementary Figures 3–8. The fact that the cyanobacterial Hyp sequences showed highly conserved residues reported to contribute importantly to the Hyp features (Supplementary Table 7 and Supplementary Figures 3–8) suggests that the process of maturation of the H_2_ase enzymes in cyanobacteria might be similar to that described in other organisms (Hansel et al., 2001; Shomura and Higuchi, 2012; Watanabe et al., 2012; Douglas et al., 2013; Tominaga et al., 2013). The *hyp* genes are either clustered together in various combinations or scattered throughout the genome without any correlations being detected with the diazotrophic ability of the strains or their habitat or their classification (Figure 4C, Supplementary Figure 1). The *hyp* genes can be classified into 11 main classes depending on their patterns of organization. The genomes in class 1 carry all the *hyp* genes in a single cluster, while those in class 2 carry five clustered *hyp* genes and one gene located in another part of the genome, for example. Many rearrangements of the *hyp* clusters have occurred during the evolution of cyanobacteria, and the number of clusters increases in the late branches of the tree. In clade h, the genes are all clustered together and show a similar pattern of organization (Figures 2, 3, Supplementary Figure 1).

### O_2_-tolerant H_2_ases

**Figure 5:**
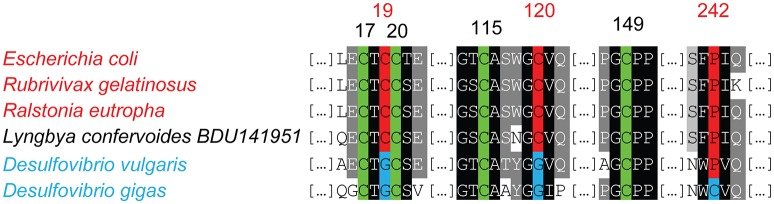
**Amino acid Alignment of the proximal cluster sequences in the small subunit of the membrane bound H_2_ases (MBH)**. The genomes harboring O_2_-tolerant MBH encoding genes, and the residues contributing importantly to O_2_-tolerance are marked in red. The genomes harboring O_2_-sensitive MBH encoding genes are indicated in blue. The cysteine residues conserved in both enzymes are shown in green.

A search for homologs of O_2_-tolerant H_2_ases encoding genes in all the cyanobacterial genomes available in the NCBI database yielded positive findings in five genomes (Table 2, Supplementary Table 8). A blast analysis using the MBH H_2_ase Hyd1 from *E.coli* (Group 1, accession number: 3UQY PDB) showed a match with a protein from *Lyngbya confervoides* BDU141951 (Chandrababunaidu et al., 2015). Multiple sequence alignments indicated that the six cysteine residues (C17, C19, C20, C115, C120, and C149 in *E. coli* HydI) involved in the coordination of the proximal [4Fe3S] as well as the proline residue (residue 242 in HydI), both of which are typical of this class of O_2_-tolerant enzymes, are conserved in the protein of *L. confervoides* BDU141951 (Figure 5).

The Hox enzyme of *Aphanocapsa montana* BDHKU210001 (Bhattacharyya et al., 2015) showed similarities with the SH H_2_ase of *R. eutropha* (Group 3d, accession numer: AAP85843.1). The HoxH, HoxY, and HoxU proteins showed 51, 50, and 45% identity, respectively, with their respective homologs in the *R. eutropha* enzyme. Homologs of the *Pyrococcus furiosus* H_2_ase SH (Group 3b) were identified in *Leptolyngbya boryana* PCC 6306, *Cyanothece* sp. PCC 7425 and *Mastigocoleus testarum* BC008. The sequences encoding the four subunits α (pf0894), β (pf0894), γ (pf0892), and δ (pf0893) showed an average rate of identity of 33% with those of *P. furiosus*. In the small subunit, the four cysteine residues serving as ligands in the coordination of the [4Fe-4S] cluster in the small subunit are conserved. In conclusion, three of the four O_2_-tolerant enzymes described so far are present in the cyanobacterial phylum. Three of the strains potentially producing these enzymes are marine (*Aphanocapsa montana* BDHKU210001, *Lyngbya confervoides* BDU141951 and *Mastigocoleus testarum* BC008), and the other two originate from freshwater environments (*Cyanothece* sp. PCC 7425, and *Leptolyngbya boryana* PCC 6306).

The maturation process of the MBH-O_2_ tolerant H_2_ase of *Ralstonia eutropha* has been shown to involve some *hox* specific genes in addition to the *hyp* genes (Bernhard et al., 1996; Schubert et al., 2007; Ludwig et al., 2009; Fritsch et al., 2011a). The peptidase specific for this enzyme is encoded by the *hoxM* gene. *hoxO* and *hoxQ* genes encode for specific chaperones and *hoxZ* for a b-type cytochrome (Bernhard et al., 1996; Schubert et al., 2007). The maturation process of the MBH-O_2_ tolerant H_2_ase of *R. eutropha* has been shown to also involve the Hox LRTV proteins (Fritsch et al., 2011b). Homologs of the *hoxZMLOQRTV* genes were searched in the genome of the cyanobacterium *Lyngbya confervoides* BDU14195, and as a control in genomes of other organisms known to harbor the MBH-O_2_ tolerant enzyme (*E. coli* (Evans et al., 2013), *Alteromonas macleodii* (Vargas et al., 2011), *Hydrogenovibrio marinus* DSM 11271 (Shomura et al., 2011), *Rubrivivax gelatinosus* (Maness et al., 2002), and *Salmonella enterica* (Bowman et al., 2014). The result of this analysis showed that while the *hoxZ, hoxM, hoxL, hoxO*, and *hoxQ* were conserved in all non-cyanobacterial genomes analyzed, only the *hoxZ*, and *hoxM* genes were identified in *Lyngbya confervoides* BDU14195 (Supplementary Table 9). The ability of this cyanobacterium to produce an active MBH-O_2_ tolerant enzyme is therefore questionable. Since the maturation process of the other O_2_-tolerant H_2_ases found in cyanobacteria has not been reported to require any specific proteins other than the Hyp, it is possible that *Aphanocapsa montana* BDHKU210001, *Cyanothece* sp. PCC 7425 and *Mastigocoleus testarum* BC008 might produce active O_2_-tolerant H_2_ases. The genome of *Leptolyngbya boryana* PCC 6306 was found to contain only the *hypAB genes*, this strains can therefore regarded as inable to build an active O_2_-tolerant H_2_ase (Supplementary Table 1).

## Discussion

The present analyses of the distribution of genes encoding H_2_ases in cyanobacterial genomes suggest that H_2_ metabolism is widely distributed among the various ecological niches that have been colonized by these organisms. H_2_ase genes and the genes encoding proteins necessary to the maturation process feature prominently in the late branching clades of the cyanobacterial tree of species, which suggests that the need for H_2_ production and/or uptake has followed the phylogenic evolution of this phylum. The fact that all the structural genes in these enzymes and their maturation process genes have been largely conserved in many cyanobacterial genomes indicates, if these genes are really expressed, that they might play an important physiological role in the bacterial strains inhabiting various environments. Considerable rates of H_2_ production by cyanobacteria have been reported to occur in microbial mats (Marshall et al., 2012), and *Microcoleus* spp has been found to be a predominant H_2_ producer in the microbial mats formed in the Elkhorn Slough estuary, Monterey Bay (Burow et al., 2012). These data further indicate that functional studies on H_2_ases in environmental strains in addition to laboratory models would greatly improve our understanding of H_2_ metabolism in this bacterial phylum. No bidirectional H_2_ase genes were detected in the genomes of open ocean strains (*Prochlorococcus* and *Synechococcus* in particular), in agreement with previous results (Barz et al., 2010). The latter study also showed that heterotrophic bacteria inhabiting this environment also lacked bidirectional H_2_ase encoding genes. The O_2_ concentration of open ocean waters measured during a period of several months was found to be above 200 μM (Emerson et al., 2002) which may not favor the contribution of the Hox enzyme to the process of H_2_ metabolism under anaerobic conditions (Khanna and Lindblad, 2015). The distribution of *hup, hox* and *nif* genes is highly variable in freshwater, hot spring and terrestrial environments (Figure 1), possibly because of the various conditions that organisms may encounter in these ecological niches.

Nineteen genomes of strains belonging to subsections I, II, III and IV contain *nif* genes but no *hup* genes (Figures 1–3 and Supplementary Table 2). In this background, one might expect the H_2_ production rate of nitrogenase to play an important role in the absence of uptake H_2_ase. The deletion of the *hupL* gene in the filamentous diazotrophic strains *Nostoc* PCC 7120 and *Nostoc* PCC 7422 has indeed been found to improve the H_2_ production (Masukawa et al., 2002; Yoshino et al., 2007). In the unicellular cyanobacterium *Cyanothece* PCC 7822, which fixes nitrogen under aerobiosis, HupL has been shown to be essential to activity of the nitrogenase in the presence of O_2_. The authors concluded that the main function of the HupSL complex in this bacterium is the protection of the nitrogenase from O_2_ (Zhang et al., 2014). The present data show that most of the strains possessing *nif* genes and lacking the uptake H_2_ase are unicellular [*Aphanocapsa montana* BDHKU210001, *Chroococcidiopsis* sp. PCC 6712, *Nodosilinea nodulosa* PCC 7104, *Synechococcus* sp. JA-2-3B'a(2–13), *Synechococcus* sp. JA-3-3Ab]. All these strains are known to undergo N_2_ fixation under anaerobic conditions (Suplementary Table 2). In future studies, it would be interesting to investigate whether the absence of the uptake H_2_ase in these strains results in high H_2_ production.

The finding that genes potentially encoding O_2_-tolerant H_2_ases are present in five cyanobacterial genomes is of great interest. Since *Lyngbya confervoides* BDU141951 genome does not contain all the accessories *hox* genes important for the maturation process of the MBH-O_2_ tolerant enzyme, and since the genome of *Leptolyngbya boryana* PCC 6306 contains only the *hypAB* genes, it is likely that these two strains are not able to produce an active O_2_-tolerant enzyme. Whether the other three cyanobacterial strains found here to possess genes encoding for O_2_-tolerant enzyme actually produce these enzymes needs to be analyzed. The possible input of theses enzymes to the physiology of these organisms in both marine and freshwater environments is an intriguing question. These enzymes are probably involved in the oxidation of H_2_, like most of their homologs in other organisms. However, in the aerobic soil bacterium *Mycobacterium smegmatis*, an O_2_-tolerant H_2_ase has been found to produce H_2_, thus enabling this organism to cope with the hypoxia occurring in its ecological niche (Berney et al., 2014). The possibility that O_2_-tolerant H_2_ase may play a similar role in cyanobacteria is a tempting hypothesis. Whether the cyanobacterial strains found to possess genes encoding for O_2_-tolerant H_2_ases could be for interest in the context of photosynthetic H_2_ production is a perspective worth exploring in the future.

## Methods

### Datasets

The genome set analyzed in this study includes 126 cyanobacterial genomes of the CyanoGeba dataset (Shih et al., 2013; Calteau et al., 2014), and genomes of *Aphanocapsa montana* BDHKU210001, *Cyanobacterium* sp. UCYN-A2, *Lyngbya confervoides* BDU141951, *Mastigocoleus testarum* BC008 which are present in the JGI database (https://img.jgi.doe.gov/cgi-bin/mer/main.cgi). In the case of H_2_ases not generally found to occur in cyanobacteria (the [FeFe] H_2_ases, and [NiFe] H_2_ases other than Hox and Hup), the analysis also included cyanobacterial genomes present in the NCBI database (https://blast.ncbi.nlm.nih.gov/Blast.cgi). The complete list of the genomes analyzed and their accession numbers is given in Supplementary Table 3.

### Database search and sequences analysis

The cyanobacterial genomes present in the databases cited above were analyzed using the sequences listed in Supplementary Table 10 as queries. The *e*-values were adapted to the legnt of the sequences analyzed. A BLASTp (Altschul et al., 1990) analysis was conducted with a specific threshold *e*-value for each protein, in order to limit the number of paralogs found and therefore to avoid false positives (Supplementary Table 8). Best Reciprocal Blast Hits method and context genomic analysis were used to discriminate false positive and to choose the best the *e*-value threshold. Sequence alignments were carried out with Clustal-W and displayed with GeneDoc (Thompson et al., 1994; Nicholas et al., 1997). Phylogenetic analysis was performed using the Neighbor-Joining (NJ) method (Saitou and Nei, 1987) implemented in Clustalw to identify eventual false positive.

### Phylogenetic analysis

The species tree was generated by concatenating 21 conserved proteins selected from the phylogenetic markers proposed for use with bacterial genome trees (Wu and Eisen, 2008). The 21 selected proteins are: DnaG, Pgk, PyrG, RplB, RplC, RplD, RplE, RplF, RplL, RplM, RplN, RplP, RplT, RpoB, RpsC, RpsE, RpsI, RpsK, RpsS, SmpB, and Tsf. The sequences of these proteins from *Anabaena variabilis* ATCC 29413 were used as queries in BlastP analyses. The genomes of *Chloroflexus auranticus* J-10, *Rhodobacter sphaeroides* 2.4.1, *Heliobacterium modesticaldum* Ice1, and *Chlorobium tepidum* TLS were used as outgroups to root the tree as previously used (Calteau et al., 2014). Multiple sequence alignments of the proteins were performed using MUSCLE 3.8.31 (Edgar, 2004). The alignments were concatenated and the phylogenetic tree was generated with PhyML 3.3.2 (BioNJalgorithm/default parameters) (Guindon et al., 2009).

## Author contributions

AL designed the study and wrote the paper, VP conducted the work, and ST participated in the analysis.

### Conflict of interest statement

The authors declare that the research was conducted in the absence of any commercial or financial relationships that could be construed as a potential conflict of interest.
