# Supplementary material for: Distribution of Hydrogenases in Cyanobacteria: A Phylum-Wide Genomic Survey
Source: Front Genet. 2016 Dec 27;7:223. doi: 10.3389/fgene.2016.00223 (PMC5186783; doi:10.3389/fgene.2016.00223)
Supplement: Supplementary file 2 [file DataSheet1.DOCX]

Supplementary Material

Article Title

Vincenzo Puggioni, Sebastien Tempel, Amel Latifi

*** Correspondence:** Amel Latifi : latifi@imm.cnrs.fr

**
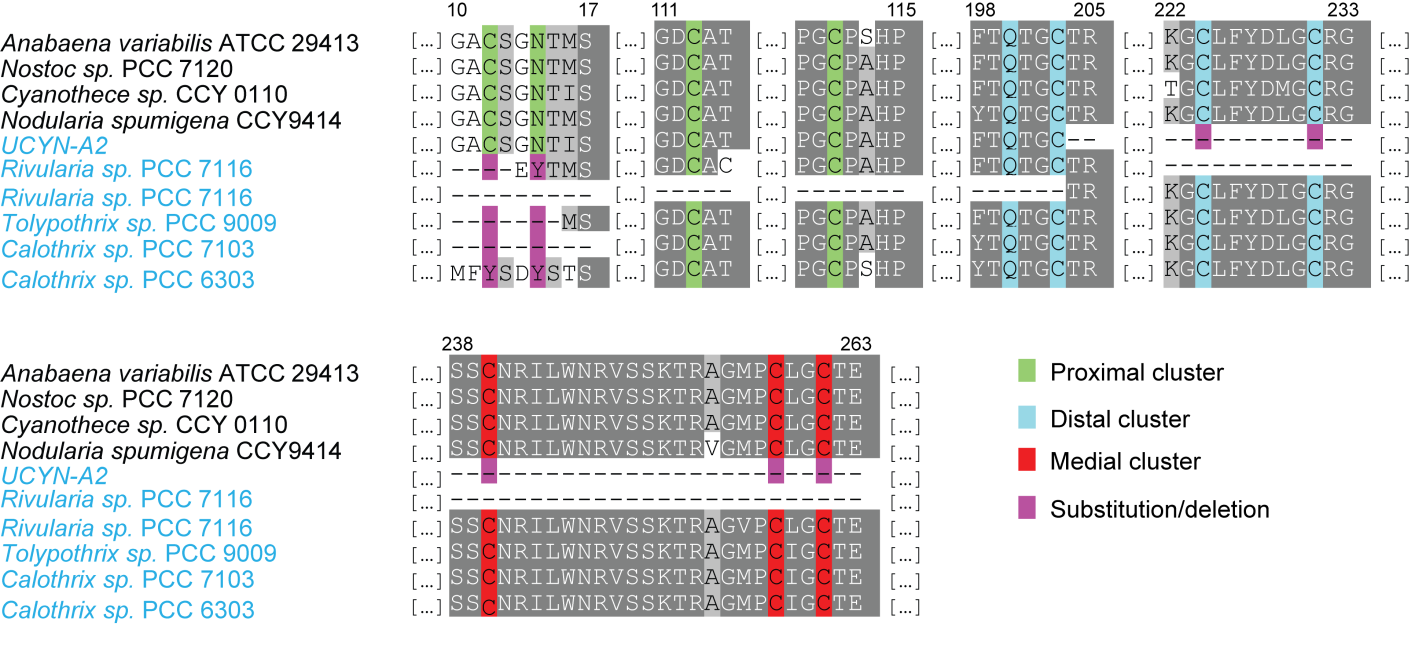
**

**Supplementary Figure 2.** Amino acid alignment of [FeS] cluster binding motifs of HupS proteins. The strains indicated in black have a well conserved HupS sequence. The strains indicated in blue present substitutions or deletions of important residues of HupS.


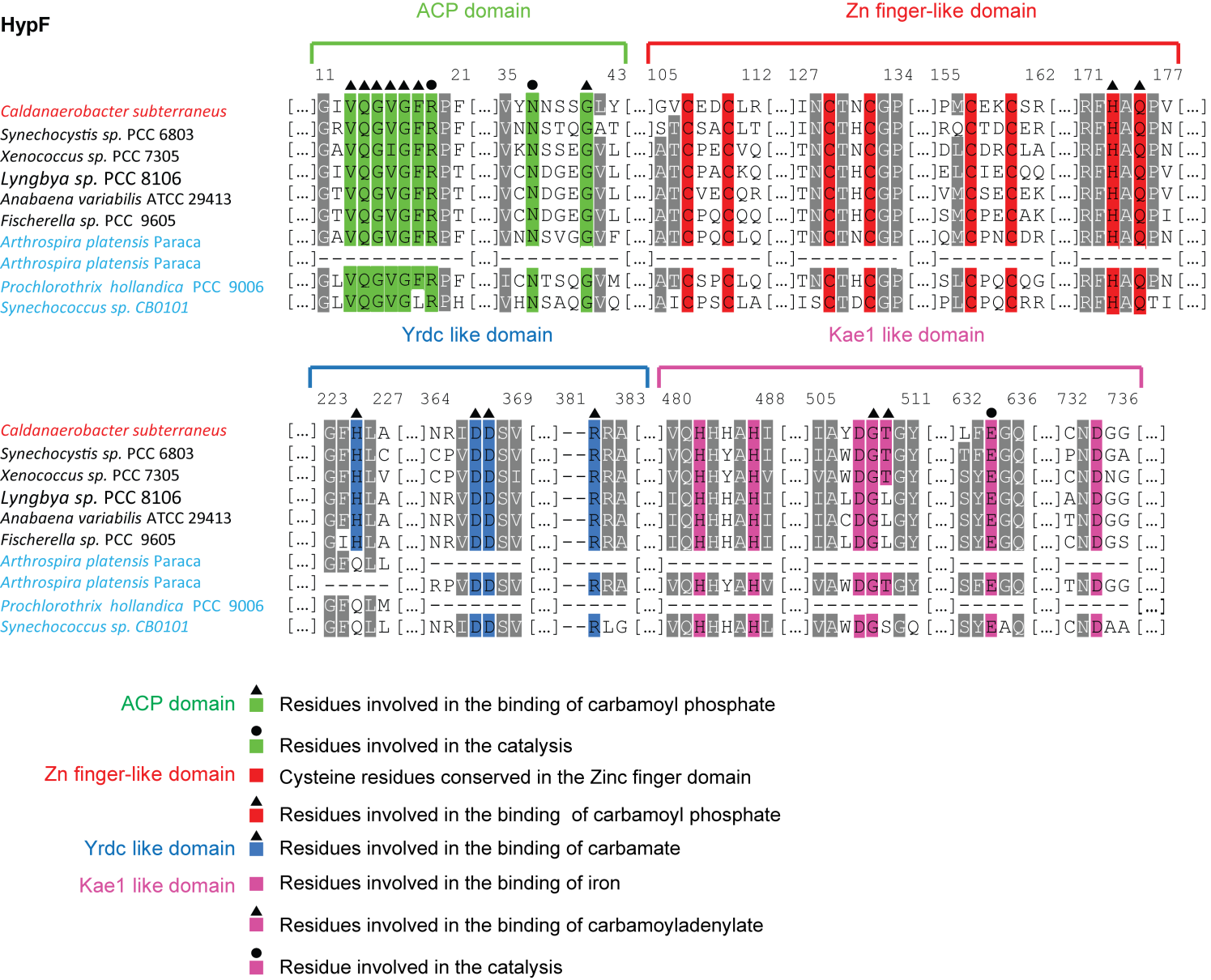


**Supplementary Figure 3.** Amino acid alignment of HypF proteins. Three no protein ligand 2(CN) and CO coordinate the iron of the binuclear center Ni-Fe of H_2_ases (active site). HypF is involved in the synthesis of CN from carbamoyl phosphate, and catalyzes the conversion of carbamoyl phosphate in carbamate, carbamoyladenylate and finally interacts with HypE to synthetize thiocyanate. (Shomura and Higuchi, 2012; Tominaga et al., 2013) The important residues have been deduced from the crystallographic structure of HypF of *Caldanaerobacter subterraneus* [PDB accession number 3VTH] (Shomura and Higuchi, 2012). They are marked in different colors and their roles are indicated at the bottom of the figure. The triangle marked the residues involved in the binding of substrates. The circle the residues involved in the catalysis. The strains indicated in black have a well conserved HypF sequence. The strains indicated in blue present important variations of the HypF sequence.


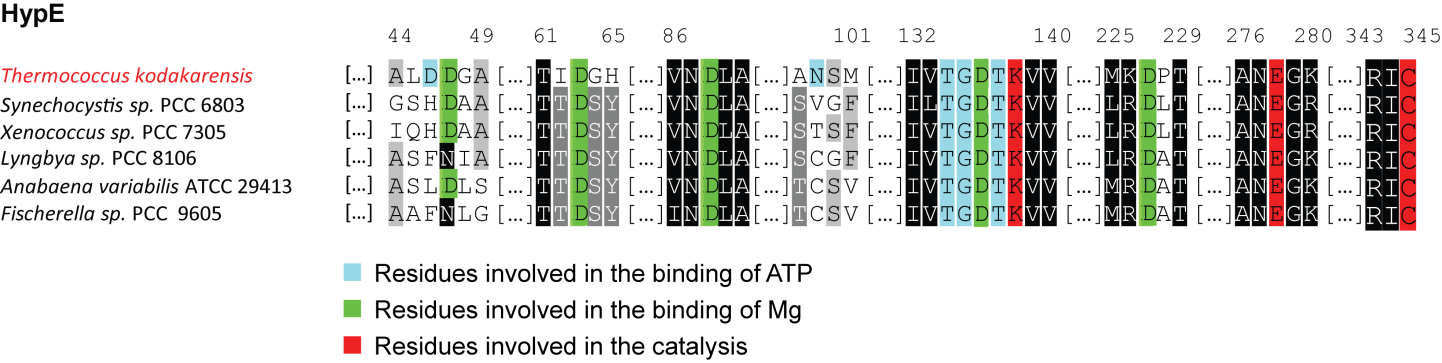


**Supplementary Figure 4.** Amino acid alignment of HypE proteins. HypE interacts with HypF to synthetize thiocyanate and with HypC and HypD to transfer the CN group to Fe atom. The important residues have been deduced crystallographic structure of HypE of *Thermococcus kodokorensis* is available [PDB accession number 3WJP (Tominaga et al., 2013)].


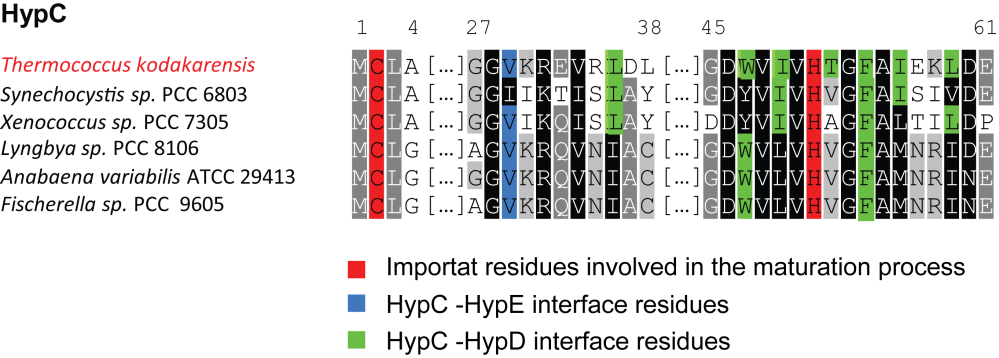


**Supplementary Figure 5.** Amino acid alignment of HypC proteins. HypC interacts with HypE and HypD. The complex HypC,D,E is responsible of cyanation of the iron center of [NiFe] hydrpgenases, and the complex HypD,E to insertion of the iron center in the large subunits. (Watanabe et al., 2007, 2012) (Watanabe et al., 2007, 2012) The crystallographic structure of HypC, and the complex Hyp CDE, and HypCD of *Thermococcus kodokorensis* are available [PDB accession number 2Z1C and 3VYS (Watanabe et al., 2007, 2012)].


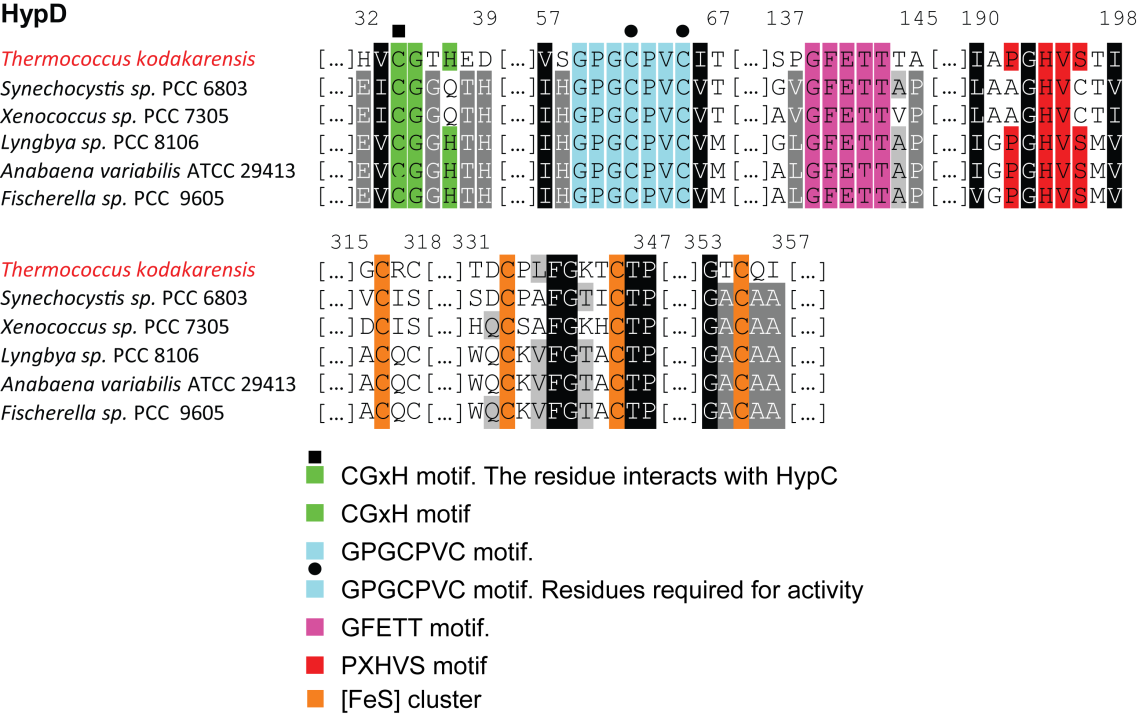


**Supplementary Figure 6.** Amino acid alignment of HypD proteins. The crystallographic structure of HypD of *Thermococcus kodokorensis* is available [PDB accession number 2Z1D (Watanabe et al., 2007)] The crystallographic structure shows the presence of a center cleft where are located the four motif indicated that probably form the HypD active site (Watanabe et al., 2007) .


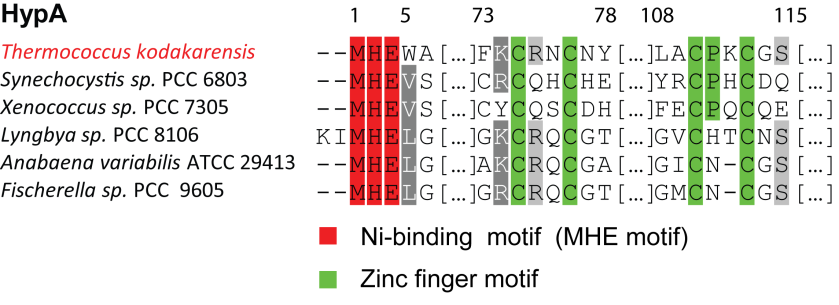


**Supplementary Figure 7.** Amino acid alignment of HypA proteins. HypA form a complex with HypB and functions as a metal chaperone in the insertion of the Ni atom into H_2_ases, last step of H_2_ases maturation. (Watanabe et al., 2009) The crystallographic structure of HypA of *Thermococcus kodokorensis* is available [PDB accession number 3VYR (Watanabe et al., 2012)].

**
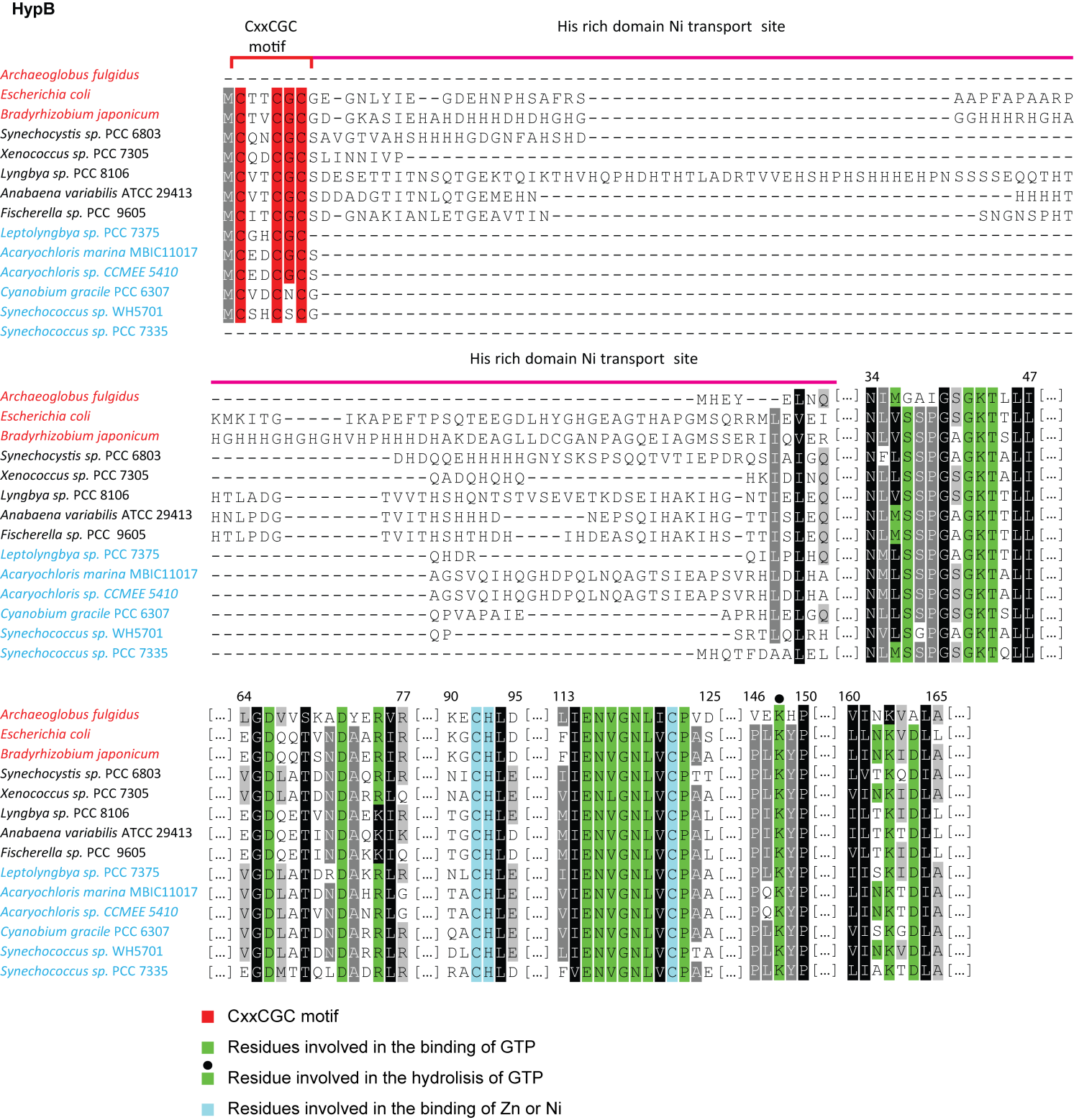
**

**Supplementary Figure 8.** Alignment of HypB homologues. The crystallographic structures of HypB of *Archaeoglobus fulgidus* is available [PDB accession number 2WSM (Chan et al., 2012), and the activity of this enzyme from *Escherichia coli* and *Bradyrhizobium japonicum* has been characterized (Douglas et al., 2013; Olson and Maier, 2000). The strains indicated in blue present substitutions or deletions of important residues of HypB.

Reference

Chan, K. H., Li, T., Wong, C. O., and Wong, K. B. (2012). Structural basis for GTP-dependent dimerization of hydrogenase maturation factor HypB. *PLoS One* 7.

Douglas, C. D., Ngu, T. T., Kaluarachchi, H., and Zamble, D. B. (2013). Metal transfer within the escherichia coli HypB-HypA complex of hydrogenase accessory proteins. *Biochemistry* 52, 6030–6039.

Olson, J. W., and Maier, R. J. (2000). Dual roles of bradyrhizobium japonicum nickelin protein in nickel storage and GTP-dependent Ni mobilization. *J. Bacteriol.* 182, 1702–1705.

Shomura, Y., and Higuchi, Y. (2012). Structural basis for the reaction mechanism of S-carbamoylation of HypE by HypF in the maturation of [NiFe]-hydrogenases. *J. Biol. Chem.* 287, 28409–28419.

Tominaga, T., Watanabe, S., Matsumi, R., Atomi, H., Imanaka, T., and Miki, K. (2013). Crystal structures of the carbamoylated and cyanated forms of HypE for [NiFe] hydrogenase maturation. *Proc. Natl. Acad. Sci. U. S. A.* 110, 20485–90. Available at: http://www.pubmedcentral.nih.gov/articlerender.fcgi?artid=3870729&tool=pmcentrez&rendertype=abstract.

Watanabe, S., Arai, T., Matsumi, R., Atomi, H., Imanaka, T., and Miki, K. (2009). Crystal Structure of HypA, a Nickel-Binding Metallochaperone for [NiFe] Hydrogenase Maturation. *J. Mol. Biol.* 394, 448–459.

Watanabe, S., Matsumi, R., Arai, T., Atomi, H., Imanaka, T., and Miki, K. (2007). Crystal Structures of [NiFe] Hydrogenase Maturation Proteins HypC, HypD, and HypE: Insights into Cyanation Reaction by Thiol Redox Signaling. *Mol. Cell* 27, 29–40.

Watanabe, S., Matsumi, R., Atomi, H., Imanaka, T., and Miki, K. (2012). Crystal structures of the HypCD complex and the HypCDE ternary complex: Transient intermediate complexes during [NiFe] hydrogenase maturation. *Structure* 20, 2124–2137.
