# Supplementary material for: Distribution of Hydrogenases in Cyanobacteria: A Phylum-Wide Genomic Survey
Source: Front Genet. 2016 Dec 27;7:223. doi: 10.3389/fgene.2016.00223 (PMC5186783; doi:10.3389/fgene.2016.00223)

Supplementary Material

Article Title

Vincenzo Puggioni, Sebastien Tempel, Amel Latifi

*** Correspondence:** Amel Latifi : latifi@imm.cnrs.fr

Supplementary Figure 1. Physical organization of hox, hup and hyp genes. The arrow of different colors represent the hox, hup and hyp. In the top of arrow is indicate the name of genes and in the bottom the position of ATG in the genome. The interruption with parallel lines indicate that the distance between genes is more than 5 kb.

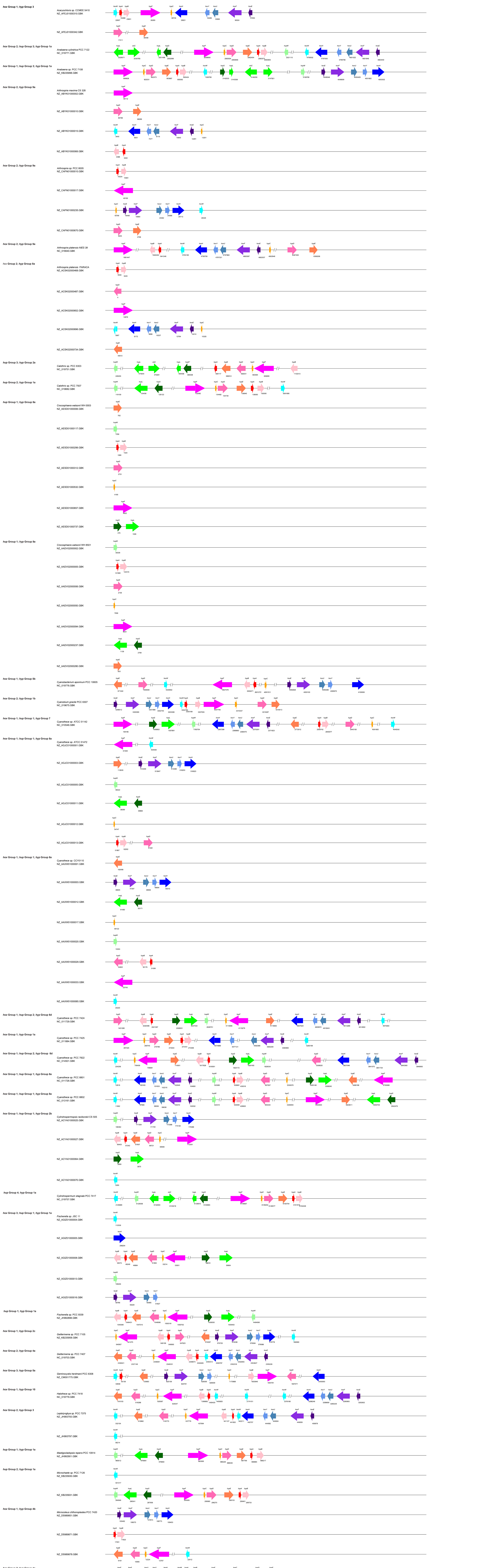

Supplement: Supplementary file 3 [file Image1.pdf]
